# Designing high-performance infrared optoelectronic materials: indium-site substitution in LiInSe_2_ with Al, Ga, Sn, and Sb

**DOI:** 10.1039/d5ra05215g

**Published:** 2025-12-04

**Authors:** Jiahuan Chen, Sichen Luo, Yuqing Yang, Yanan Zhang, Suwen Han, Pengfei Lu, Chunlian Xiong, Yue Cheng, Changcheng Chen, Xiaoning Guan

**Affiliations:** a International School, Beijing University of Posts and Telecommunications Beijing 100876 China guanxn@bupt.edu.cn; b School of Integrated Circuits, Beijing University of Posts and Telecommunications Beijing 100876 China; c School of Science, Xi'an University of Architecture and Technology Xi'an 710055 China

## Abstract

In this study, we employed first-principles calculations based on density functional theory to systematically evaluate the impact of substituting Al, Ga, Sn and Sb atoms into LiInSe_2_ crystals with *R*3*m* and *I*4_1_/*amd* space groups. By adjusting the doping ratios of these elements, we analyzed their effects on the optoelectronic properties of LiInSe_2_. The results reveal that Al doping reduces the formation energy in the *I*4_1_/*amd* structure, indicating easier synthesis under conventional conditions. Moreover, Al incorporation increases the bandgap, thereby raising the excitation energy required for electronic transitions. In contrast, Ga, Sn and Sb dopants tend to increase the formation energy while narrowing the bandgap. Further analysis identified four effective doping pathways in the *R*3*m* structure, all exhibiting potential for enhanced optoelectronic performance. Notably, Sb doping-despite its higher formation energy and reduced bandgap compared to the intrinsic structure-significantly enhances the optical absorption response in 1.65–3.00 eV. The structural modifications induced by Al, Ga, and Sb doping contribute to improved crystal stability and broadened spectral response, underscoring the strong potential of these materials for infrared detector applications.

## Abstract 2

First-principles calculations reveal that targeted doping (Al, Ga, Sb) effectively tunes LiInSe_2_’s band gap and enhances mid-wave infrared absorption, with Sb in the *R*3*m* space group exhibiting exceptional optoelectronic performance.

## Graphical abstract

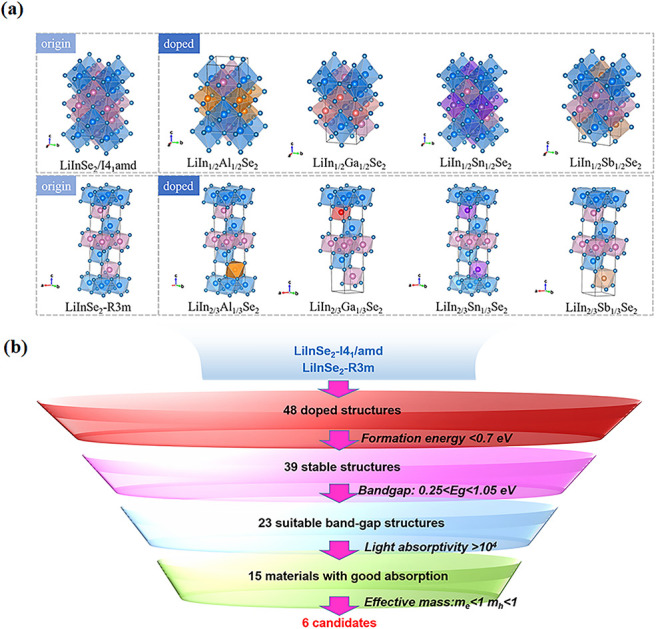

## 1 Introduction

Infrared detectors, as core optoelectronic devices, are widely used in fields such as military applications, medical diagnostics, and environmental monitoring. Current mainstream infrared detectors can be categorized into two types: cooled detectors (*e.g.*, InSb, HgCdTe(MCT), and Type-II superlattice detectors(T2SL)) and uncooled detectors (*e.g.*, vanadium oxide and amorphous silicon). Each type presents its own limitations in performance. Although InSb-based detectors are mature in applications, their narrow bandgap, fixed wavelength response, and low operating temperature hinder their suitability for engineering applications.^1^ MCT detectors suffer from issues such as poor material uniformity, unstable interfaces, and complex epitaxial growth and post-processing.^2–5^ T2SL offer advantages such as tunable bandgaps, low Auger recombination rates, large effective electron masses, and good material uniformity; however, their actual device performance has not yet met theoretical expectations.^6–11^ Although uncooled infrared detectors are cost-effective, compact, and lightweight,^12,13^ and thus attractive for low-performance applications, they are still inferior to cooled detectors in terms of sensitivity, noise level, and detection range.^14,15^ To overcome the bottlenecks in traditional infrared materials-such as poor bandgap tunability, limited device stability,^16^ and complex fabrication processes-it is urgent to explore novel infrared detection materials with outstanding optoelectronic performance. Given the potential applications of chalcopyrite-type compounds in various infrared domains,^17^ we hypothesize that LiInSe_2_ crystals could serve as promising alternative materials for infrared detection.^18–21^ Therefore, this work adopts a theoretical design approach, utilizing elemental substitution and doping to investigate and optimize the LiInSe_2_ system for infrared detection applications. Owing to its unique electronic, thermal, and structural characteristics,^22,23^ LiInSe_2_ exhibits excellent optical absorption properties. It offers high transmittance in commonly used infrared bands,^24^ a broad transparency window, significant birefringence, a high laser damage threshold, and a low two-photon absorption coefficient,^25^ making it a highly regarded candidate in the field of infrared detection.^26,27^ Nevertheless, reports on LiInSe_2_ remain scarce in the literature,^28^ likely due to its electronic structure instability, limited optical performance, and intrinsic defects that cause optical losses and lead to laser-induced damage,^29–31^ which impede its further application in infrared optoelectronic devices.^32^ Moreover, the difficulty in obtaining indium^33^ restricts its large-scale production. Still, alternative strategies such as forming heterojunctions, tuning the elemental composition, and introducing foreign dopants^34^ remain viable avenues for exploration. Since there are few existing studies on doping in LiInSe_2_,^35^ we selected several metal atoms for substitute doping in the LiInSe_2_ crystal structure. Database analysis suggests that the introduction of Ga and Al could enhance structural stability and photoelectric conversion efficiency;^36,37^ Sb doping may broaden the infrared transparency range and enhance nonlinear optical effects;^38^ Sn doping is expected to regulate carrier concentration^39^ and potentially improve optoelectronic properties.

### 1.1 Why Sn behaves differently

While Sn is often invoked to tune carrier concentration, our calculations reveal that In → Sn substitution in LiInSe_2_ tends to collapse the band gap and yield semi-metallic states. This behavior originates from (i) the valence mismatch between Sn^2+/4+^ and In^3+^ that promotes compensating defects or off-stoichiometry, and (ii) strong hybridization between Sn-5s/5p and Se-4p states that shifts the band edges and introduces band crossings. As a result, even when the formation energy can be small or negative in *R*3*m* for certain loadings, the resulting electronic structure is not suitable for MWIR photodetectors that require a finite gap. This motivates a focused discussion on Sn in the Results section. A key factor in the practical use of LiInSe_2_ lies in its good carrier mobility, which supports efficient photoelectric conversion. Based on this, we conducted further research to reveal how various metal dopants influence its electronic structure and optical properties. Research on the optoelectronic properties of LiInSe_2_ generally focuses on the *I*4_1_/*amd* and *R*3*m* space groups, as they are more stable and easier to control under standard conditions. In our design, Al, Ga, Sb, and Sn atoms were introduced to partially replace atoms in LiInSe_2_, resulting in four new compound types: LiAlInSe_2_, LiGaInSe_2_, LiSbInSe_2_ and LiSnInSe_2_. In this study, we used DFT-based first-principles calculations to investigate the intrinsic structures of LiInSe_2_ under both space groups, as well as the doped structures with Al, Ga, Sb, and Sn. After structural optimization, we calculated properties such as formation energy, bandgap, density of states, and optical absorption. The results show that in the *I*4_1_/*amd* space group, Al doping reduces the formation energy and thus improves structural stability, while Ga, Sb and Sn doping increase the formation energy. In the *R*3*m* space group, the formation energy of the Al-doped structure is −0.9938 eV, the lowest among all configurations, indicating the most stable structure. Sb doping follows, and Ga doping yields a positive but relatively small formation energy. In terms of bandgap, the intrinsic *I*4_1_/*amd* structure has a relatively small bandgap of 0.3957 eV. Al doping increases the bandgap, whereas Ga, Sb and Sn doping decrease it. Sb and Sn doping even lead to gap closure, indicating that while Al doping enhances structural stability, it also raises the excitation energy required for electronic transitions. For the *R*3*m* space group, the intrinsic bandgap is 0.8283 eV. Doping with Al, Ga, and Sb (in two configurations) reduces the bandgap, which may be beneficial for improving optoelectronic performance. Regarding optical absorption, Al doping enhances the absorption of the *I*4_1_/*amd* structure in the 0.1–0.8 eV range. In the *R*3*m* structure, Sb doping significantly boosts absorption in the 1.65–3 eV range, thereby expanding the potential application range of LiInSe_2_.

## 2 Computational details

All calculations were based on the DFT method and employed the Projector Augmented-Wave (PAW) method. The electron exchange–correlation interaction was described using the Generalized Gradient Approximation (GGA) of the Perdew–Burke–Ernzerhof (PBE) functional. The plane-wave kinetic energy cutoff was set to 1.3 times the maximum ENMAX value among all pseudopotentials used in the system, and was validated through convergence tests to ensure the reliability of the computational results. The convergence criterion for structural optimization was set such that the force on each atom was less than 1 × 10^−6^ eV Å^−1^. For *k*-point sampling, a Gamma-centered 4 × 4 × 4 uniform *k*-point mesh was used for structural optimization, total energy calculations, and density of states (DOS) to ensure efficient sampling of the Brillouin zone. The band structure calculations adopted the high-symmetry path *k*-point scheme, sampling along high-symmetry points in the Brillouin zone to obtain a clear band structure.^40^ The carrier effective mass was computed using the finite-difference method, with its numerical parameters validated through convergence testing. The selected *k*-point step size (Δ*k* = 0.015 Å^−1^) ensures robust numerical stability of the results.

**Fig. 1:**
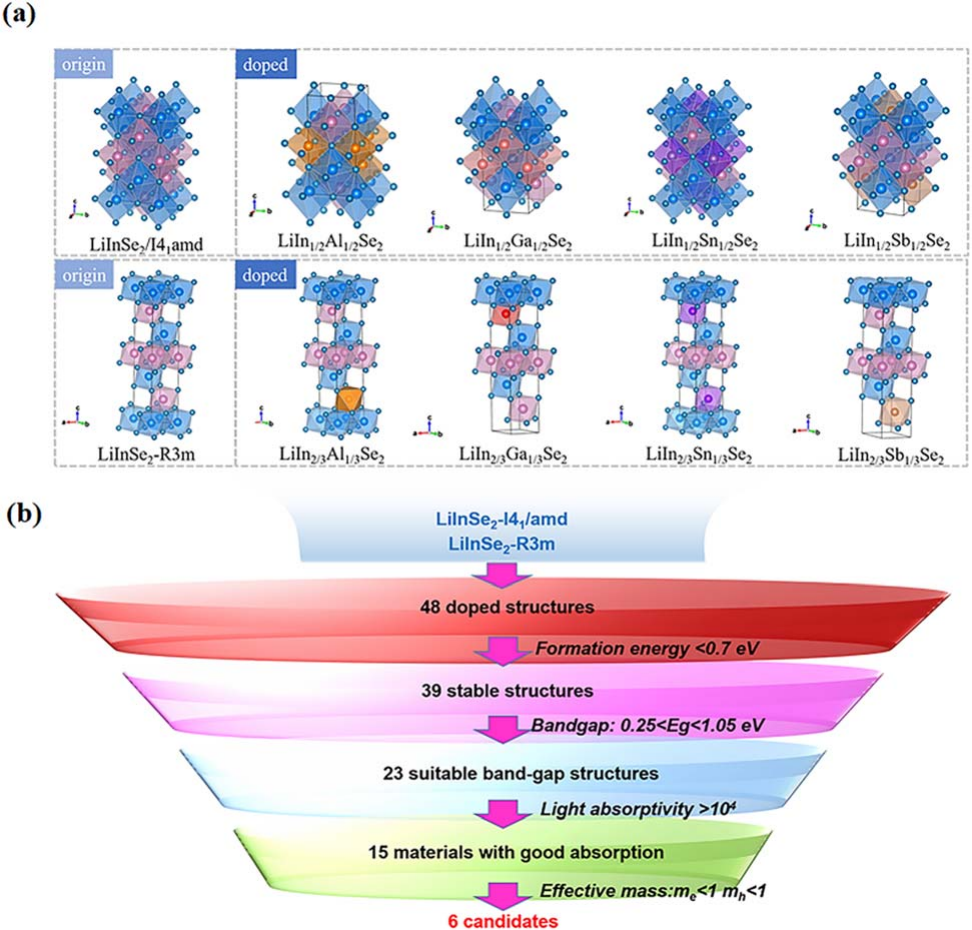
(a) Structural models of LiInSe_2_ with space groups *I*4_1_/*amd* and *R*3*m*, along with the corresponding doped structures incorporating Al/Ga/Sn/Sb elements. The models labeled as origin represent the pristine structures, while those labeled as doped correspond to the four doped configurations. (b) Screening process of the doped structures used in this work.

The *I*4_1_/*amd* space group of LiInSe_2_ corresponds to a layered tetragonal system, while the *R*3*m* space group belongs to the rhombohedral system. Their structure is shown in Fig. 1. As shown in Tables 1 and 2, after optimization, the lattice constants were determined to be *a* = *b* = 5.6589 Å, *c* = 11.3044 Å, *α* = *β* = *γ* = 90°, and *a* = 3.9588 Å, *b* = 3.4284 Å, *c* = 19.5983 Å, *α* = *β* = 90°, *γ* = 120°, respectively. To visualize the coordination of LiInSe_2_ atoms, a 1 × 1 × 2 supercell was constructed, comprising 16 atoms (Li_4_In_4_Se_8_) in the *I*4_1_/*amd* space group and 12 atoms (Li_3_In_3_Se_6_) in the *R*3*m* space group, both adopting a chalcopyrite structure.^41^

**Table 1:** Lattice constants (*a*, *b*, *c*), electron effective mass (*m*_e_), and hole effective mass (*m*_h_) of pristine and doped LiInSe_2_ with space group *I*4_1_/*amd*. The “—” indicates structures that did not meet the screening criteria and thus were not calculated further

*I*4_1_/*amd*	*a* (Å)	*b* (Å)	*c* (Å)	*m*_e_	*m*_h_
LiIn_1/2_Al_1/2_Se_2_	5.5261	5.4640	11.3246	0.334	0.903
LiIn_1/2_Ga_1/2_Se_2_	5.5583	5.5807	11.1850	0.675	0.889
LiIn_1/2_Sn_1/2_Se_2_	5.6310	5.6204	11.3881	—	—
LiIn_1/2_Sb_1/2_Se_2_	5.6439	5.6368	11.5232	—	—
LiInSe_2_	5.6589	5.6589	11.3044	0.376	0.478

**Table 2:** Lattice constants (*a*, *b*, *c*), electron effective mass (*m*_e_), and hole effective mass (*m*_h_) of pristine and doped LiInSe_2_ with space group *R*3*m*. The “—” indicates structures that did not meet the screening criteria and thus were not calculated further

*R*3*m*	*a* (Å)	*b* (Å)	*c* (Å)	*m*_e_	*m*_h_
Liln_1/3_Al_2/3_Se_2_	3.7786	3.2723	19.3063	0.265	0.202
Liln_2/3_Ga_1/3_Se_2_	3.8967	3.3746	19.5002	0.929	0.829
Liln_2/3_Sn_1/3_Se_2_	3.9615	3.4308	19.7025	—	—
LiIn_2/3_Sb_1/3_Se_2_	3.9588	3.4284	19.6052	0.651	0.225
LiInSe_2_	3.9588	3.4284	19.5983	0.470	0.786

In this study, the in atomic content in the LiInSe_2_ crystal structure was modified by substituting in atoms with Al, Ga, Sn, and Sb atoms, resulting in the formula LiIn_1−*x*_M_*x*_Se_2_, where M represents different dopant atoms.^42^ For the *I*4_1_/*amd* and *R*3*m* space groups, the doping concentrations were set to 50% (*I*4_1_/*amd*), 33% (*R*3*m*), and 66% (*R*3*m*), respectively. Meanwhile, six different doping configurations were considered for the *I*4_1_/*amd* structure (labeled as *ab*, *ac*, *etc.*), and three different doping configurations were considered for the *R*3*m* structure (labeled as 1, 2, and 3).

To screen high-*l* stability. Then, in order to identify semiconductor structures with appropriate bandgap values suitable for MWIR property studies, 23 structures with bandgaps larger than 0.25 eV and smaller than 1.05 eV were further selected. To identify materials with strong optical absorption, 15 structures exhibiting absorption coefficients greater than 10^4^ cm^−1^ were then screened out. Finally, based on the calculated carrier effective masses, 6 structures were chosen for presentation and detailed analysis in the following study.

## 3 Results and discussion

The relative stability of all possible doped defect configurations in LiInSe_2_ was systematically evaluated through formation energy calculations. For Al-, Ga-, Sn-, and Sb-doped defect models, the formation energy *E*_form_ was calculated as follows:^43^

1 *E*_form_ = *E*_doped_ − *E*_pristine_ + *E*_M_ − *E*_*x*_

In this equation, *E*_doped_ and *E*_pristine_ represent the total energies of the doped and pristine systems, respectively. *E*_M_ denotes the reference state energy of the doping atom, while *E*_*x*_ corresponds to the reference state energy of the substituted atom.

**Fig. 2:**
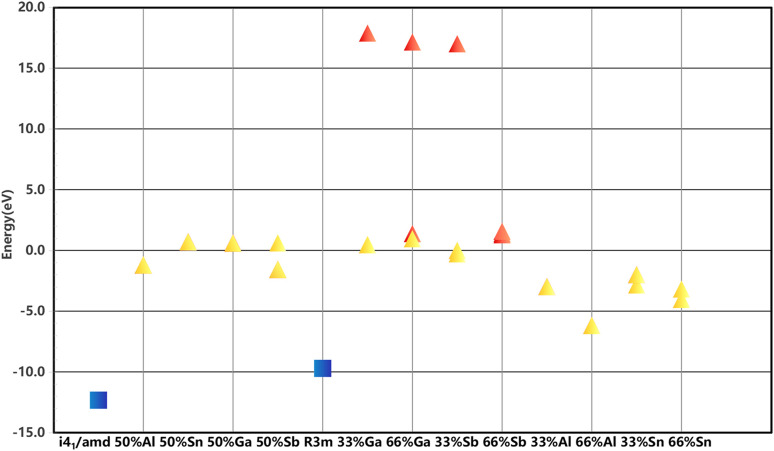
Formation energies of all structures are represented by scatter plots. Blue squares denote the two pristine structures, yellow triangles indicate configurations meeting the screening criteria, and red triangles represent those failing the criteria. Structures are grouped by space group (*I*4_1_/*amd* and *R*3*m*).

The sign and magnitude of the formation energy reflect the energetic cost of the doping process and the stability of the doped structure. A negative formation energy indicates a thermodynamically favorable doping process, while a smaller formation energy suggests higher stability of the doped configuration. Fig. 2 illustrates the distribution of formation energies for the four dopants in both *I*4_1_/*amd* and *R*3*m* space groups.

Subsequently, we calculated the bandgap energies of these structures for further screening, and the selected results based on our established criteria are summarized in Table 4.

Our calculations revealed that the electronic and optical properties of different doping sites in the *I*4_1_/*amd* space group exhibited remarkable similarity. Consequently, we identified six representative structures for in-depth analysis. These include two from the *I*4_1_/*amd* space group (LiIn_1/2_Al_1/2_Se_2_-bc* and LiIn_1/2_Ga_1/2_Se_2_-ab*) and four from the *R*3*m* space group (LiIn_2/3_Ga_1/3_Se_2_-3*, LiSb_1/3_In_2/3_Se_2_-2*, LiSb_1/3_In_2/3_Se_2_-1*, and LiAl_1/3_In_2/3_Se_2_-1*).

### 3.1 Band structure and band gap analysis

**Fig. 3:**
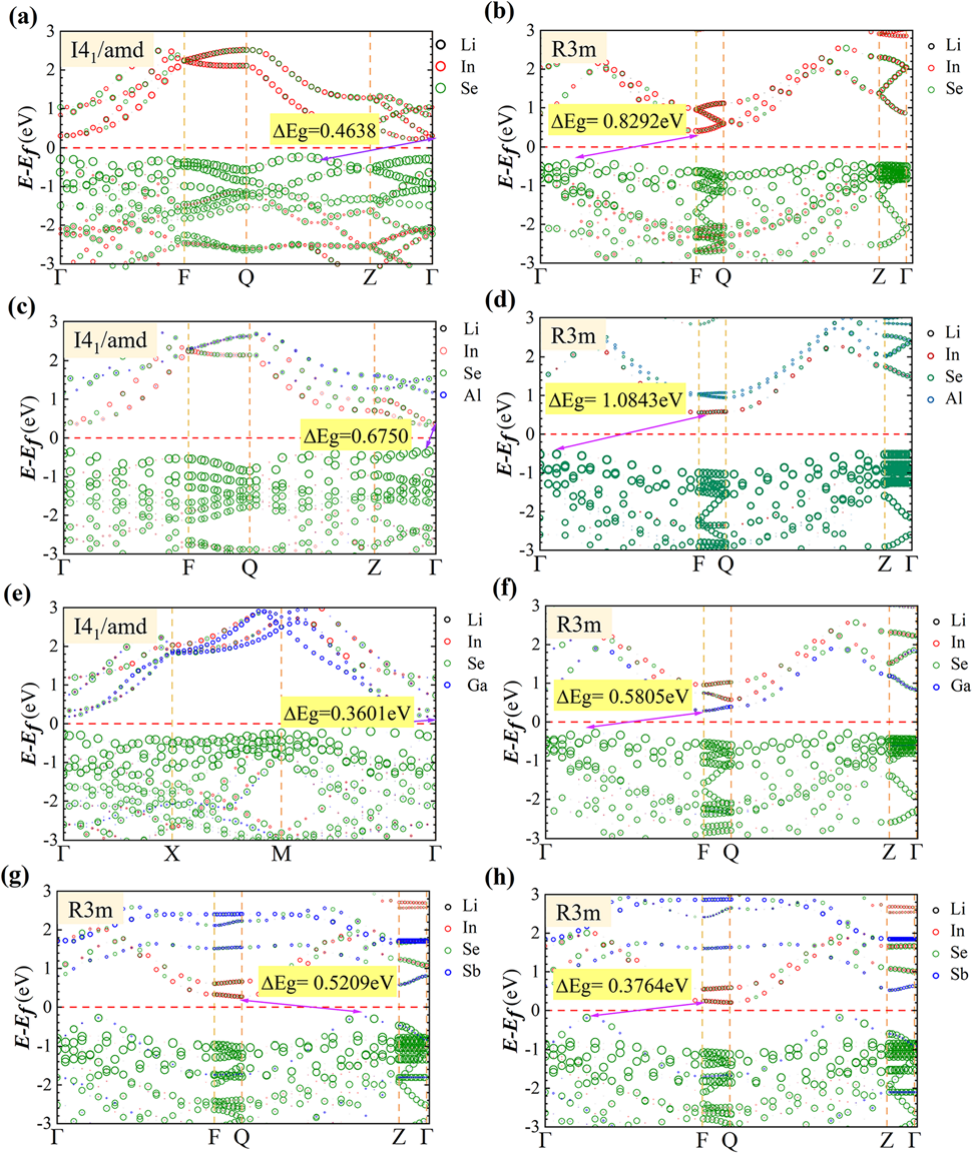
Band structure diagrams of the calculated structures for: (a) pristine LiInSe_2_ in the *I*4_1_/*amd* phase, (b) pristine LiInSe_2_ in the *R*3*m* phase, (c) *I*4_1_/*amd* with 50% Al substitution at In sites, (d) *R*3*m* with 33% Sb doping at Wyckoff position 1, (e) *I*4_1_/*amd* with 50% Ga substitution at In sites, (f) *R*3*m* with 33% Sb doping at Wyckoff position 2, (g) *R*3*m* with 33% Ga substitution at In sites, and (h) *R*3*m* with 33% Al substitution at In sites. The Fermi level is marked by red dashed lines in all panels.

We systematically investigated the electronic structure and bonding characteristics of LiInXSe_2_ (X = Al, Ga, Sn, Sb) using the GGA-PBE functional,^44^ which revealed fundamental properties of these materials. Fig. 3 presents the calculated band structures obtained using the PBE functional, comprising six screened doped configurations and two pristine systems.

The band structure calculations reveal that all LiInXSe_2_ configurations exhibit distinct semiconductor characteristics: the *I*4_1_/*amd* phase displays an indirect Γ–Z bandgap of 0.46 eV, while the *R*3*m* phase shows a direct Γ–Γ bandgap of 0.83 eV, demonstrating significant phase-dependent electronic structure variations.

Notably, elemental substitution induces significant bandgap modulation effects: Al doping increases the *I*4_1_/*amd* bandgap to 0.67 eV, while Ga doping reduces the bandgaps to 0.36 eV (*I*4_1_/*amd*) and 0.58 eV (*R*3*m*). This strong contribution of Ga 4p states near the CBM aligns with previous DFT predictions showing bandgap narrowing in Ga-doped LiInSe_2_.^45,46^ Sb doping decreases the *R*3*m* bandgap to 0.52 eV and 0.38 eV.

### 3.2 Why Sn doping drives semi-metallicity

Our band structure calculations reveal that In → Sn substitution in LiInSe_2_ leads to near-gap closure and semi-metallic electronic states. This distinct behavior arises from the aliovalent nature of Sn, where Sn^2+^/Sn^4+^ valence mismatch with In^3+^ promotes compensating defects and shifts the Fermi level into conduction states. In addition, the strong hybridization between Sn 5s/5p and Se 4p orbitals introduces band crossings and localized states near the Fermi level. As a result, even when some Sn-doped *R*3*m* configurations show relatively low formation energies, as shown in Table 3, their electronic structures are unsuitable for mid-wave infrared detection applications that require a finite bandgap.

**Table 3:** Formation energies (in eV) of doped and pristine LiInSe_2_ configurations in *I*4_1_/*amd* and *R*3*m* space groups

*I*4_1_/*amd*	*E* (eV)	*R*3*m*	*E* (eV)
LiInSe_2_	−12.3269	LiInSe_2_	−9.7102
Liln_1/2_Al_1/2_Se_2_-ab	−1.2050	Liln_2/3_Ga_1/3_Se_2_-1	0.4684
Liln_1/2_Al_1/2_Se_2_-ac	−1.1740	Liln_2/3_Ga_1/3_Se_2_-3	0.4763
Liln_1/2_Al_1/2_Se_2_-ad	−1.2050	LiIn_2/3_Sb_1/3_Se_2_-1	−0.2886
Liln_1/2_Al_1/2_Se_2_-bc	−1.2050	LiIn_2/3_Sb_1/3_Se_2_-3	−0.0013
Liln_1/2_Al_1/2_Se_2_-bd	−1.1761	LiIn_2/3_Al_1/3_Se_2_-1	−2.9831
Liln_1/2_Al_1/2_Se_2_-cd	−1.2050	LiIn_2/3_Al_1/3_Se_2_-2	−2.9831
Liln_1/2_Sn_1/2_Se_2_-ab	0.7044	LiIn_2/3_Al_1/3_Se_2_-3	−2.9831
Liln_1/2_Sn_1/2_Se_2_-ac	0.7014	LiIn_1/3_Al_2/3_Se_2_-1	−6.1739
Liln_1/2_Sn_1/2_Se_2_-ad	0.7044	LiIn_1/3_Al_2/3_Se_2_-2	−6.1789
Liln_1/2_Sn_1/2_Se_2_-bc	0.7044	LiIn_1/3_Al_2/3_Se_2_-3	−6.1789
Liln_1/2_Sn_1/2_Se_2_-bd	0.7014	LiIn_2/3_Sn_1/3_Se_2_-1	−2.8425
Liln_1/2_Sn_1/2_Se_2_-cd	0.7044	LiIn_2/3_Sn_1/3_Se_2_-2	−1.9963
Liln_1/2_Ga_1/2_Se_2_-ab	0.5930	LiIn_2/3_Sn_1/3_Se_2_-3	−1.9963
Liln_1/2_Ga_1/2_Se_2_-ac	0.6275	LiIn_1/3_Sn_2/3_Se_2_-1	−4.0279
Liln_1/2_Ga_1/2_Se_2_-ad	0.6102	LiIn_1/3_Sn_2/3_Se_2_-2	−3.1819
Liln_1/2_Ga_1/2_Se_2_-bc	0.6104	LiIn_1/3_Sn_2/3_Se_2_-3	−4.0279
Liln_1/2_Ga_1/2_Se_2_-bd	0.6275		
Liln_1/2_Ga_1/2_Se_2_-cd	0.6102		
Liln_1/2_Sb_1/2_Se_2_-ab	0.6235		
Liln_1/2_Sb_1/2_Se_2_-ac	−1.5303		
Liln_1/2_Sb_1/2_Se_2_-ad	−1.5769		
Liln_1/2_Sb_1/2_Se_2_-bc	−1.5769		
Liln_1/2_Sb_1/2_Se_2_-bd	−1.5303		
Liln_1/2_Sb_1/2_Se_2_-cd	−1.5769		

**Table 4:** Calculated bandgap energies (in eV) for pristine and doped LiInSe_2_ configurations in *I*4_1_/*amd* and *R*3*m* space groups. The doping concentrations are indicated by subscripts (*e.g.*, LiIn_1−*x*_M_*x*_Se_2_ with *x* = 1/2, 1/3, or 2/3). Configurations marked with an asterisk (*) were selected for in-depth analysis

*I*4_1_/*amd*	Bandgap (eV)	*R*3*m*	Bandgap (eV)
LiInSe_2_	0.4638	LiInSe_2_	0.8283
Liln_1/2_Al_1/2_Se_2_-ab	0.5606	Liln_2/3_Ga_1/3_Se_2_-1	0.5763
Liln_1/2_Al_1/2_Se_2_-ac	0.5784	Liln_2/3_Ga_1/3_Se_2_-3*	0.5805
Liln_1/2_Al_1/2_Se_2_-ad	0.5572	LiIn_2/3_Sb_1/3_Se_2_-1*	0.5209
Liln_1/2_Al_1/2_Se_2_-bc*	0.6767	LiIn_2/3_Sb_1/3_Se_2_-2*	0.3764
Liln_1/2_Al_1/2_Se_2_-bd	0.5831	LiIn_2/3_Al_1/3_Se_2_-1*	1.0843
Liln_1/2_Al_1/2_Se_2_-cd	0.5591		
Liln_1/2_Ga_1/2_Se_2_-ab*	0.3601		
Liln_1/2_Ga_1/2_Se_2_-ac	0.6275		
Liln_1/2_Ga_1/2_Se_2_-ad	0.6102		
Liln_1/2_Ga_1/2_Se_2_-bc	0.6105		
Liln_1/2_Ga_1/2_Se_2_-bd	0.6275		
Liln_1/2_Ga_1/2_Se_2_-cd	0.6102		

We attribute these effects primarily to varying degrees of hybridization between the p-orbitals of dopant elements (Al, Ga, Sb) and Se 4p states. The results demonstrate that Al doping effectively increases the bandgap, while Ga and Sb doping reduces it, with Sb doping at site 2 showing the most significant bandgap reduction effect. The band gap is smaller than that of Li doping substitution, which is conducive to improving the absorption capacity of mid-wave infrared.^47^

### 3.3 PDOS contribution analysis

**Fig. 4:**
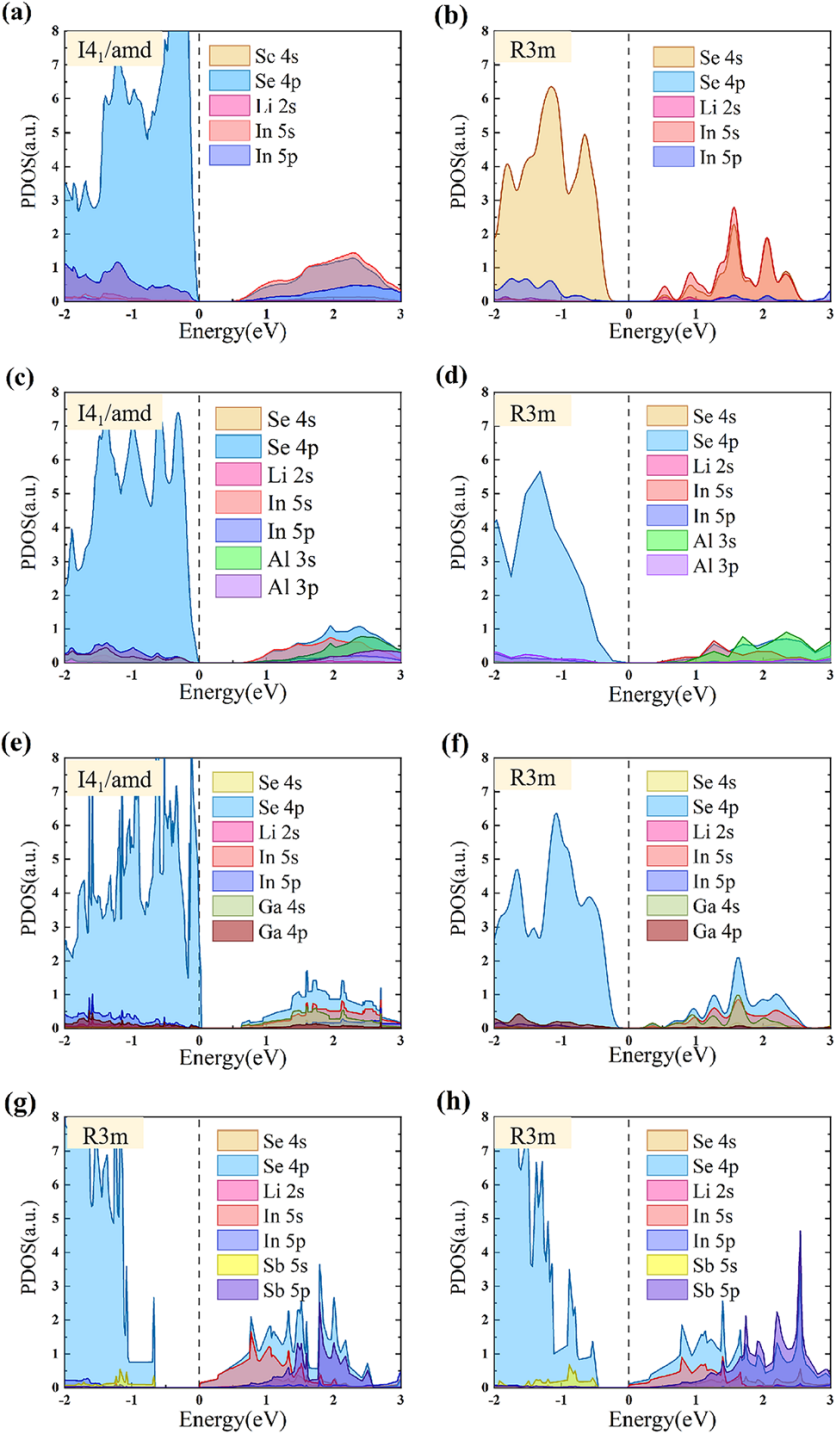
(a) Presents pristine *I*4_1_/*amd* structure, (b) pristine *R*3*m* structure, (c) *I*4_1_/*amd* with 50% Al doping, (d) *R*3*m* with 33% Sb doping at site 1, (e) *I*4_1_/*amd* with 50% Ga doping, (f) *R*3*m* with 33% Sb doping at site 2, (g) *R*3*m* with 33% Ga doping, and (h) *R*3*m* with 33% Al doping.

Fig. 4 presents the calculated density of states diagrams, revealing that the electronic states between −2 and 3 eV exhibit distinct orbital distribution characteristics. The top valence band region (−1 eV to Fermi level) is dominated by strongly hybridized Se 4p and in 5p orbital (about 70% contribution), with minimal Li-s orbital participation (about 5%), demonstrating the pronounced covalent character of In–Se bonds.^48^ In the lower energy region (−2 to −1 eV), the DOS is primarily composed of Se 4p states with minor contributions from in 5s and Li 2p orbitals, consistent with reported behavior in isostructural LiGaSe_2_. The bottom conduction band states mainly consist of Li 2 s and in 5s orbitals, while doping introduces significant Ga 4p/Al 3p contributions (about 35%), confirming their critical role in bandgap modulation. Notably, Ga and Sb orbitals exhibit greater influence near band edges compared to Al, with Sb-doped systems showing localized Sb 5p-derived impurity states near 1 eV that significantly modify the electronic structure. Our calculated redshift in the absorption edge is in line with prior experimental observations on stoichiometry variations in LiInXSe_2_, which revealed similar spectral modifications due to intrinsic defects.^49^ While dopant states contribute minimally to the valence band, they participate substantially in the conduction band, with about 35% enhancement in Ga/Al p-orbital contributions particularly highlighting their bandgap engineering capability. These results quantitatively establish the orbital-resolved electronic structure modifications induced by various doping schemes. Compared with the *Pna*2_1_ space group, the high contribution of Se at the −2–0 eV energy level is consistent.^50^

Our analysis reveals significant differences in doping-induced projected density of states(PDOS) variations between the *I*4_1_/*amd* and *R*3*m* space groups of LiInXSe_2_, uncovering crucial structure–property relationships. In the *I*4_1_/*amd* configuration, Al doping causes notable bandgap widening, primarily due to reduced contributions from Al 3p orbitals near the conduction band minimum (CBM) compared to the native atoms. This leads to decreased electronic state density around the Fermi level, enhancing the material's wide-bandgap semiconductor characteristics-this finding fully consistent with band structure calculations showing CBM upshift, suggesting excellent potential for applications requiring larger bandgaps. In contrast, Ga doping exhibits the opposite effect, with its 4p orbitals making substantial contributions near the CBM that significantly increase the Fermi-level state density, thereby facilitating electron excitation to the conduction band and reducing the bandgap. This phenomenon is particularly prominent in the *R*3*m* space group, where the enhanced conductivity makes Ga-doped systems especially suitable for applications demanding high electrical conductivity.

Notably, Sb doping induces distinctive electronic structure modifications in both space groups, generating additional impurity states near band edges while simultaneously reducing the bandgap. Our analysis reveals enhanced contributions from Sb 5p orbitals at the valence band maximum compared to native atoms, suggesting improved hole transport properties and potential p-type conductivity. Particularly in the *R*3*m* structure, Sb doping causes remarkable alterations in the Fermi-level state density and introduces mid-gap states, which may significantly influence carrier recombination dynamics.

Comparative analysis between space groups demonstrates that the *R*3*m* configuration exhibits greater sensitivity to doping, manifested through more pronounced PDOS variations near the Fermi level, while the *I*4_1_/*amd* structure maintains relatively stable electronic distribution with stronger electron localization characteristics. This feature most prominent in Al-doped systems. These systematic PDOS variations corroborate the band structure calculations, providing crucial doping-specific electronic structure modulation strategies for optimizing the optoelectronic performance and charge transport properties of LiInSe_2_.

### 3.4 Analysis of light absorption capacity and absorption peak

In this study, the absorption peaks of the pristine LiInSe_2_–*I*4_1_/*amd* structure appear at 1.5 eV and 2.8 eV in the *x*, *y* direction, and at 1.2 eV and 2.5 eV in the *z* direction, while the pristine *R*3*m* configuration shows absorption peaks at 1.6 eV and 2.5 eV in the *x*, *y* direction, and at 1.6 eV in the *z* direction.

**Fig. 5:**
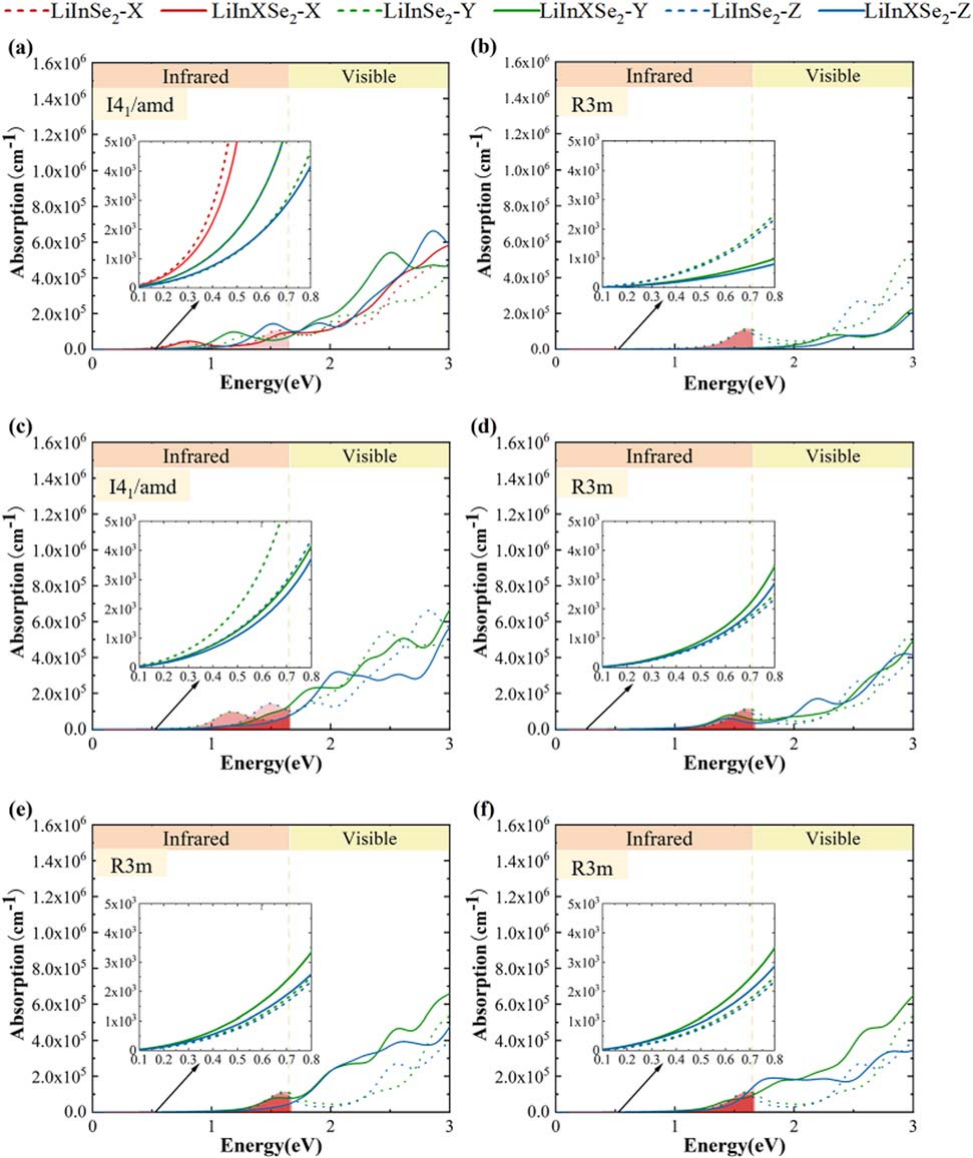
Optical absorption spectra of all selected structures: (a) 50% Al-doped *I*4_1_/*amd* phase; (b) 33% Al-doped *R*3*m* phase; (c) 50% Ga-doped *I*4_1_/*amd* phase; (d) 33% Ga-doped *R*3*m* phase; (e) 33% Sb-doped *R*3*m* phase at site 1; (f) 33% Sb-doped *R*3*m* phase at site 2. The insets show magnified views of the 0.1–0.8 eV range.

Upon doping, significant changes are observed. As shown in Fig. 5, in the Al-doped *I*4_1_/*amd* structure, the original 1.5 eV peak in the *x*, *y* direction red shifts to 0.9 eV, and the 2.8 eV peak nearly disappears. In the *z* direction, the 1.2 eV peak also red shifts to 0.9 eV, while the 2.5 eV peak blue shifts to 3.0 eV. For Ga doping in *I*4_1_/*amd*, the 1.5 eV peak shifts to 2.0 eV and the 2.8 eV peak moves beyond 3.0 eV in the *x*, *y* direction, whereas the 1.2 eV peak in the *z* direction almost vanishes, and the 2.5 eV peak slightly blue shifts to 2.6 eV.

In the doped *R*3*m* structure, Al doping results in the disappearance of most peaks around 1.6 eV and 2.8 eV in both *y* and *z* directions. For Ga doping in *R*3*m*, the peaks in the *x*, *y* direction slightly red shift to 1.5 eV and 2.3 eV, and a similar red shift is observed for the 1.6 eV peak in the *z* direction. In the Sb-1 doped structure, the 1.6 eV peak in the *x*, *y* direction nearly disappears, while the 2.5 eV peak remains largely unchanged; similarly, the 1.6 eV absorption peak in the *z* direction almost vanishes. In contrast, for the Sb-2 doped structure, the 1.6 eV peak in the *x*, *y* direction blue shifts to 1.8 eV, and the 2.5 eV peak slightly shifts to around 2.7 eV, with no significant absorption response observed at 1.6 eV in the *z* direction.^51^

In this study, we analyzed the optical absorption properties of different configurations of LiInSe_2_ and its doped structures. In the *x*, *y* direction, Al-doped structures did not exhibit a significant enhancement in absorption, while Ga-doped and Sb-doped structures showed noticeable improvements. In the *z* direction, Al-doped structures presented relatively minor changes, whereas Ga-doped and Sb-doped structures exhibited substantial enhancement in optical absorption.

For the *I*4_1_/*amd* phase, Al doping caused a red shift of the absorption peaks, enhancing absorption in the low-energy region. This behavior can be attributed to the introduction of new hybrid states near the CBM by Al^3+^ ions, resulting in bandgap narrowing and increased low-energy absorption. In contrast, Ga doping led to a blue shift of the absorption peaks. For the *R*3*m* phase, Al doping induced an overall blue shift of the absorption peaks, while Ga doping had minimal effect. Sb doping significantly enhanced absorption in the 1.65–3 eV range.

This behavior is consistent with recent findings by Zhang *et al.*, who demonstrated that suppressing deep-level defects in LiInSe_2_ significantly enhances its MIR laser emission efficiency. This may be due to Al^3+^ raising the CBM and lowering the VBM, thereby increasing the optical transition onset. The weak hybridization between Ga 4s and Se 4p orbitals introduced no significant new electronic states. However, compared with the Ga-doped LiGaTe system reported in previous literature,^52^ our Ga-doped structure still exhibits reasonably good absorption performance. In contrast, the Sb 5p orbitals contributed more substantially to both the CBM and VBM, introducing new states and enabling more transitions, thus enhancing light absorption.

In summary, the doped structures generally exhibited improved optical absorption properties. For the *I*4_1_/*amd* phase, doping significantly enhanced the low-energy absorption performance. For the *R*3*m* phase structures with reduced bandgaps, the optical absorption was also notably improved.

We compared the application characteristics of LiInSe_2_, InSb, and HgCdTe in the field of infrared detectors. InSb is a narrow-bandgap semiconductor with relatively fixed intrinsic absorption characteristics in the MWIR spectrum (3–5 µm), renowned for its high quantum efficiency and electron mobility.^53^ Although InSb has made progress in band engineering, such as *n*Bn structures that can raise its operating temperature,^54^ its band properties are fundamentally less tunable than those of doped LiInSe_2_. HgCdTe, on the other hand, boasts a unique advantage with a continuously tunable bandgap by altering its composition (*x*), allowing it to address the infrared detection needs of multiple atmospheric windows in the 1–14 µm range.^54^ However, HgCdTe's drawbacks include material growth instability and the difficulty of eliminating the non-ideal valence band offset, which severely limits its practical performanc.^55^ In summary, LiInSe_2_'s unique advantage lies in its optical absorption properties, which can be precisely tuned through doping. For example, doping with Al can cause the absorption peak to red shift to 0.9 eV, while doping with Sb can enhance absorption in the 1.65–3 eV range, providing tunable optical characteristics that could be explored in nonlinear optics and laser-related studies.

## 4 Conclusion

In this study, the effects of substituting In atoms with four different elements (Al, Ga, Sn, and Sb) on the crystal structure, electronic properties, and optical responses of LiInSe_2_ were systematically investigated in two space groups (*I*4_1_/*amd* and *R*3*m*) using first-principles calculations based on density functional theory (DFT). Interestingly, such space-group-dependent behavior was also observed in the LiIn_*x*_Ag_1−*x*_Se_2_ system, where a gradual transition from *I*4̄2*d* to *Pna*2_1_ led to notable variations in optical responses.^56^ Among 48 substitutional structures, six representative structures within specific property ranges were selected to study how atomic doping can modulate the optoelectronic performance of the material. The results show that among the four dopants, Al exhibits relatively low formation energy and increases the band gap while significantly enhancing the optical absorption above 2 eV, making it suitable for optoelectronic applications that require a wide band gap. Compared with AgInSe_2_, LiInSe_2_ offers superior optical nonlinearity and a broader infrared (IR) transparency window, as confirmed by Reshak and Brik's comparative study.^57^ Sn doping results in a negative band gap, altering the material's semiconducting nature. Ga and Sb doping significantly reduce the band gap, improve carrier transport properties, and exhibit better absorption performance in the mid-wave infrared (MWIR) region. Particularly in the *R*3*m* space group, all doped structures except for Sn demonstrate enhanced optoelectronic performance, with Sb doping showing especially remarkable improvements. Through a comprehensive analysis of band structures, density of states, and absorption spectra, this study confirms the effectiveness of doping strategies in optimizing the performance of novel MWIR materials. In conclusion, this work not only provides theoretical support for designing MWIR photodetector materials and expands the potential application scope of LiInSe_2_ in optoelectronics but also lays a foundation for future experimental fabrication and device optimization. Further exploration of co-doping schemes and structural optimization strategies may lead to even greater breakthroughs in material performance.

## Author contributions

Chen J.: software validation formal analysis investigation data curation writing – original draft writing – review & editing visualization Luo S.: methodology software validation formal analysis investigation data curation writing – original draft writing – review & editing funding acquisition Yang Y.: software validation formal analysis investigation data curation writing – original draft writing – review & editing Zhang Y.: resources writing – review & editing visualization Han S.: resources writing – review & editing visualization Xiong C.: writing – review & editing Cheng Y.: writing – review & editing Cheng C.: writing – review & editing Guan X.: conceptualization, methodology, resources, writing – review & editing, visualization, supervision, project administration Lu P.: conceptualization supervision.

## Conflicts of interest

There are no conflicts to declare.

## Data Availability

The data that support the findings of this study are available on reasonable request from the corresponding author (e-mail: guanxn@bupt.edu.cn). The data are not publicly available due to privacy and/or ethical restrictions.
