# Correction: Designing high-performance infrared optoelectronic materials: indium-site substitution in LiInSe_2_ with Al, Ga, Sn, and Sb

**DOI:** 10.1039/d6ra90033j

**Published:** 2026-04-01

**Authors:** Jiahuan Chen, Sichen Luo, Yuqing Yang, Yanan Zhang, Suwen Han, Pengfei Lu, Chunlian Xiong, Yue Cheng, Changcheng Chen, Xiaoning Guan

**Affiliations:** a International School, Beijing University of Posts and Telecommunications Beijing 100876 China guanxn@bupt.edu.cn; b School of Integrated Circuits, Beijing University of Posts and Telecommunications Beijing 100876 China; c School of Science, Xi'an University of Architecture and Technology Xi'an 710055 China

## Abstract

Correction for ‘Designing high-performance infrared optoelectronic materials: indium-site substitution in LiInSe_2_ with Al, Ga, Sn, and Sb’ by Jiahuan Chen *et al.*, *RSC Adv.*, 2025, **15**, 47981–47988, https://doi.org/10.1039/D5RA05215G.

The authors regret that the affiliations for Yanan Zhang and Suwen Han were incorrectly shown in the original manuscript. The correct list of affiliations is as shown here.

The Royal Society of Chemistry apologises for these errors and any consequent inconvenience to authors and readers.

